# Pulmonary manifestation of immunoglobulin G4-related disease in a 7-year-old immunodeficient boy with Epstein-Barr virus infection: a case report

**DOI:** 10.1186/s13052-016-0269-0

**Published:** 2016-06-08

**Authors:** Aleksandra Szczawinska-Poplonyk, Irena Wojsyk-Banaszak, Katarzyna Jonczyk-Potoczna, Anna Breborowicz

**Affiliations:** Department of Pediatric Pneumonology, Allergology and Clinical Immunology, Karol Marcinkowski University of Medical Sciences, Szpitalna Street 27/33, 60-572 Poznan, Poland; Department of Pediatric Radiology, Karol Marcinkowski University of Medical Sciences, Poznan, Poland

**Keywords:** Case report, IgG4, Immunodeficiency, Tumor, Lungs, Children

## Abstract

**Background:**

Immunoglobulin G4-related disease (IgG4-RD) is a multiorgan fibroinflammatory condition with lymphoplasmacytic infiltrates containing abundant IgG4-positive plasma cells. The immunopathogenesis of the disease and the potential role of triggering autoantigens or infectious factors have not been clearly defined. Immunoglobulin G4-related lung disease is a new and emerging condition in pediatric patients and to date, there have been only two reports regarding pulmonary manifestation of IgG4-RD in children recently published. This is the first report of IgG4-related lung disease in an immunodeficient child with Epstein-Barr virus infection.

**Case presentation:**

We report on the case of a 7-year old atopic boy who was hospitalized with an initial clinical and radiological diagnosis of pneumonia, positive Epstein-Barr virus (EBV)-DNA in the blood and defective adaptive immunity. The lung CT showed a consolidated mass lesion adjacent to the posterior wall of the chest and the diaphragm. The child underwent surgical resection of the tumor, and the histologic examination of the lung specimens revealed lymphoplasmacytic infiltrates with fibrosis and vasculitis correlating with IgG4-related lung disease. Subsequent monitoring of the patient with lung CT, pulmonary function tests and IgG4 levels did not show signs of active disease.

**Conclusions:**

The diagnosis of IgG4-related lung disease in children is challenging because of its rarity, nonspecific symptomatology and heterogeneous morphological manifestations. Further studies are required in children with pulmonary presentation of IgG4-RD to better understand pathogenesis of this condition, possible immunological or infectious triggering factors, and finally, to determine pediatric patient-targeted therapeutic interventions.

**Electronic supplementary material:**

The online version of this article (doi:10.1186/s13052-016-0269-0) contains supplementary material, which is available to authorized users.

## Background

Immunoglobulin G4-related disease (IgG4-RD) is a systemic fibroinflammatory condition with elevated serum IgG4 concentration in most cases and multiorgan involvement. It is characterized by lymphoplasmacytic infiltrates with fibrosis, phlebitis and an accumulation of IgG4-expressing plasma cells at the affected sites. IgG4-related disease is an emerging and increasingly recognized condition with a broad spectrum of clinicopathologic features and various clinical phenotypes, including manifestations in the digestive system, the exocrine and endocrine glands, kidneys, soft tissues, the skin, the cardiovascular system and the respiratory tract [1, 2]. The immunopathogenesis of the disease, the potential role of triggering autoantigens or infectious factors and the defective innate or adaptive immune response have not been clearly defined [3]. The demography of IgG4-RD was initially characteristically limited to adult patients, with 90 % of them being aged >50 years and with pancreatitis as a leading manifestation [4], but synchronous or metachronous development of fibroinflammatory lesions in other organs led to the concept of systemic IgG4-RD. Lesions associated with IgG4-RD can also be localized in the respiratory tract, in the lung parenchyma, airways, pleura, and in the mediastinum [5, 6]. IgG4-RD is an unrecognized disease and to date, there have been only two reports regarding IgG4-related lung disease in children which have been recently published [7, 8]. To our knowledge, this is the first report of a pediatric patient with IgG4-related lung disease coexisting with defective T-cell and B-cell-related adaptive immunity and EBV infection.

## Case presentation

We report on the case of a 7-year-old boy referred to our pediatric pneumonology, allergology and clinical immunology department because of chest pain and fever. He had the history of a blunt injury to the right side of his chest while he was training in martial arts a few days before admission. He did not manifest either cough, dyspnea, problems with breathing or hemoptysis nor had night sweats, weight loss or chronic loss of appetite.

His past medical history was significant for allergic rhinitis and a few episodes of bronchitis which have been treated with inhaled bronchodilators (see Additional file 1). The family history was non-contributory.

**Fig. 1:**
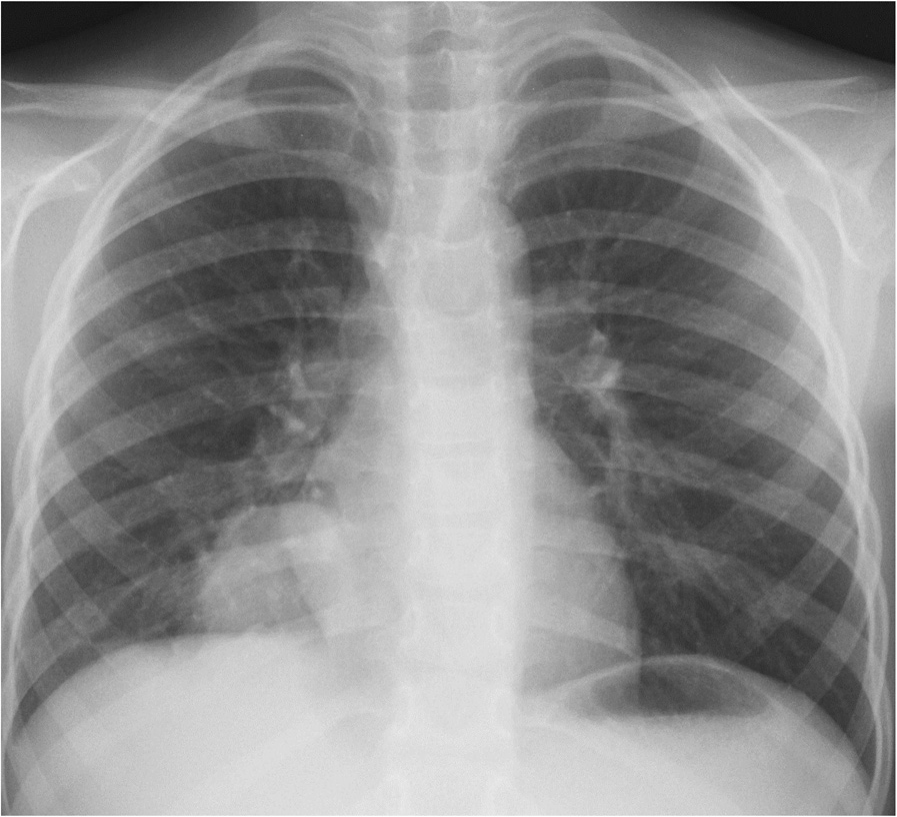
CXR showing a round lesion in the right lower lobe consistent with alveolar infiltrations, localized in segments 7, 8, and 9 and 7,0 × 5,0 cm in size (length x width) with air bronchogram

**Fig. 2:**
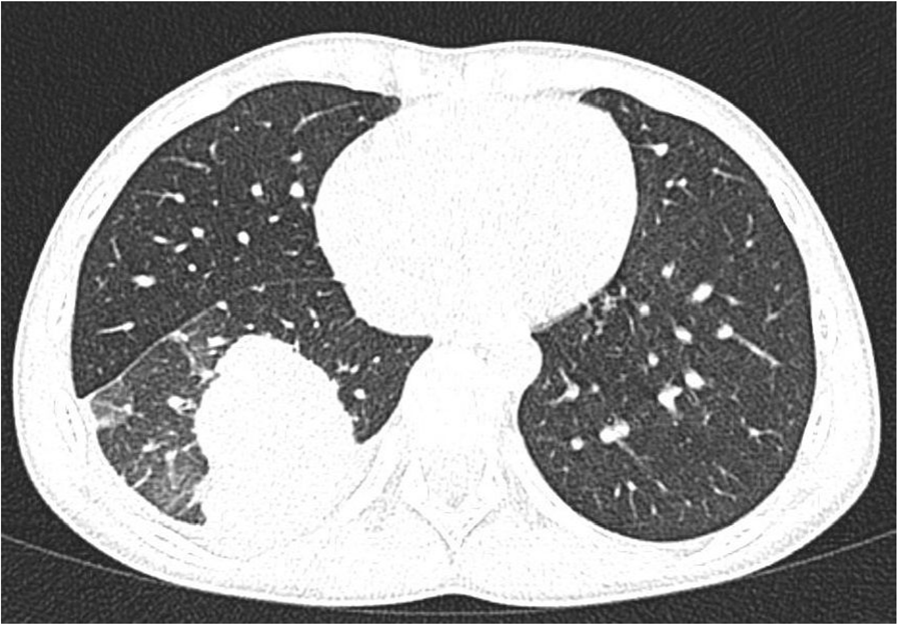
Chest CT (axial view) showing a well-defined solid mass in the right lower lobe with calcifications, surrounded by ground-glass haze and contiguous to the posterior chest wall, the diaphragm and the lung hilum

**Fig. 3:**
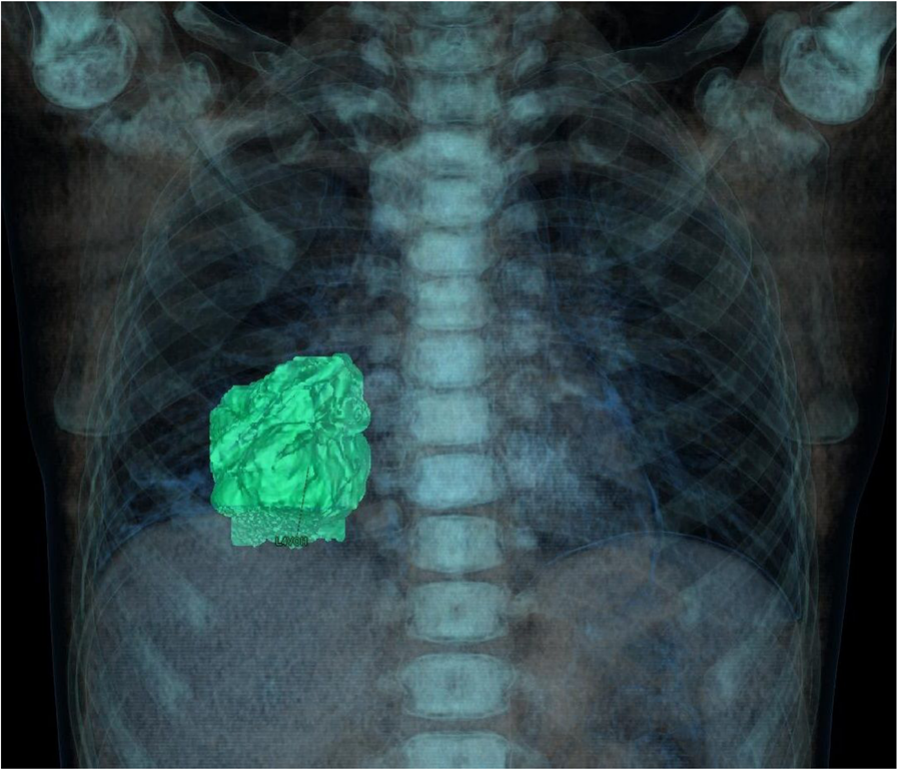
Chest 3D CT scan, volume rendering technique (VRT), evaluating the tumor mass

On admission the patient was febrile with body temperature 38,6 °C, anxious, with respiratory distress, tachypnea, shallow respiration, dyspnea, and intercostal retractions. The physical examination revealed submandibular and cervical lymphadenopathy, purulent secretions on the erythematous posterior pharyngeal wall, chronic tonsillitis, and on lung auscultation, diminished breath sounds over the right lower lobe at the back and at the front axillary line, starting from the fifth intercostal space. The chest X-ray (CXR) showed a round lesion in the right lower lobe consistent with parenchymal infiltrations (Fig. 1) and hypoechogenic areas in the lung ultrasound examination. Assuming this to be pneumonia, the patient was administered combined intravenous antibiotic therapy with cefotaxime and clarithromycin, but despite improvement in his general condition, the consolidation in the right lung did not resolve. A chest computed tomography (CT) was then conducted and revealed a solid, well-defined mass in the right lower lobe with calcifications, surrounded by ground-glass haze and contiguous to the posterior chest wall, the diaphragm and the lung hilum (Figs. 2 and 3). Inferring this to be a neoplastic lesion the VATS (video-assisted thoracoscopy) lung biopsy was performed which showed an inflammatory pseudotumor. A broad differential diagnosis was performed, including laboratory tests (summarized in Table 1). Since the results of the lung biopsy were inconclusive, the tumor was surgically removed and a histologic examination of the lung specimens was carried out, showing features of IgG4-RD. Microscopic analysis of the lung specimens revealed a focal lesion with central sclerosing, infiltrating alveolar septa, surrounding bronchovascular bundles and vascular walls. In the areas of the massive storiform fibrosis, disseminated abundant lymphoplasmacytic infiltrations with sparse eosinophils, obliterating vascular lumen were observable. Detailed description of the histopathologic findingss, including microscopic analysis and immunohistochemical (IHC) reactions, is displayed in Table 2. As IgG4-RD is potentially a relapsing condition, close monitoring of the disease activity is of utmost importance. At present, after a six-month post-surgery period, the lung CT shows only a limited fibrotic strand. IgG4 serum levels, both pre and postoperative, remain normal (42,16 mg/dl vs 42,59 mg/dl, respectively). During follow-up visits the child remains symptom-free and without further treatment. A PET (positron emission tomography) scan has been considered for the next follow-up visit to exclude a systemic manifestation of IgG4-RD and eventually to give implications for immunosuppressive therapy.

**Table 1:** Laboratory tests included in the differential diagnosis of the pulmonary nodular lesion

Laboratory tests	Results
Inflammatory markers	White blood count 10,61 G/l, lymphocytic predominance in differential (57 %) C-reactive protein (CRP) increased 2,46 mg/dl
Biochemistry	Serum activity of amylase 23 IU/l, lipase 3 IU/l, aminotransferases normal
Infections	Specific serum IgM and IgG antibodies against Mycoplasma pneumoniae, Toxoplasma gondii, hepatitis B and C viruses (HBV, HCV), cytomegalovirus (CMV) negative Epstein-Barr virus (EBV)-DNA in blood positive (1400 copies/ml) Galactomannan (Aspergillus antigen) in serum negative QuantiFERON TB negative Sputum culture: Staphylococcus aureus MSSA
Neoplastic markers	Serum alpha-fetoprotein (AFP) 1,1 ng/ml, carcinoembryonic antigen (CEA) 0,24 ng/ml, beta-human chorionic gonadotropin (β-HCG) <2,39 mIU/ml not elevated Secretion of catecholamines in 24-hour urine collection normal: vanillylmandelic acid (VMA) 7,3 mg/24 hrs, dopamine 190,6 mcg/24 hrs
Allergy	Total serum IgE elevated 660 kU/l Allergen-specific IgE against airborn and food allergens: horse 0,7 kU/l (1 class), wormwood 0,7 kU/l (1 class)
Immunology	Serum immunoglobulin (Ig) G and M normal (989 and 86 mg/dl, respectively), IgA increased 197 mg/dl Complement in serum normal C3 135 mg/dl, C4 23 mg/dl IgG subclasses normal: IgG1 617,90 mg/dl, IgG2 237,20 mg/dl, IgG3 54,87 mg/dl, IgG4 42,16 mg/dl Flow cytometric peripheral blood immunophenotyping: lymphocytes 5300 cells (50 %), increased absolute count and relative value of CD8+ T cells (2760 cells/mm3, 50 %) and decreased of CD4+ T cells (718 cells/mm3, 13 %), defective B-cell maturation: decrease of the absolute count and relative value of switched memory CD19 + CD27 + sIgD- B cells (39 cells/mm3, 7,9 %) and of non-switched memory CD19 + CD27 + sIgD+ B cells (14 cells/mm3, 2,9 %), plasmablasts CD19 + CD38hisIgM- normal (13 cells/mm3, 2,6 %)
Autoimmunization	ANA antibodies against nRNP/Sm, Sm, SS-A, SS-B, Ro-52, Scl-70, PM-Scl, Jo-1, CENP B, PCNA, AMA M2, dsDNA, nucleosomes, histones, ribosomal protein P negative ANCA antibodies against protease-3, myeloperoxidase, elastase, lactoferrin, catepsin G, BPI negative

**Table 2:** Histopathologic features of the lung biopsy

Histopathology	Features
Microscopic analysis	Marked CD138+ plasma cells and lymphocytes
	Infiltrations by IgG4-positive plasma cells with IgG4:IgG >40 %
	IgG4 cells per HPF >10
	Storiform fibrosis
	Obliterative vascular lesions (EvG staining)
IHC reactions	Alk 19 (−)
	β-catenin (−)
	desmin (−)
	vimentin (+)
	SMA (−)
	EMA (+)
	WT1 (−)
	AFP (−)
	Myogenin (−)
	MyoD1 (−)
	Glipican 1 (−)
	CD34 (−)
Other stainings	Trichrom-Masson (+)
	Amyloid (−)

## Conclusions

Immunoglobulin G4-related lung disease manifests with a spectrum of nonspecific symptoms such as cough, dyspnea, fever, and hemoptysis which along with pulmonary consolidations may mimic pneumonia or pleural effusion [5, 6, 9]. In children, the rarity of IgG4-RD is the next challenge in establishing an accurate diagnosis [7, 8] and the disease remains unrecognized. However, the awareness of IgG4-RD among pediatricians is increasing and more and more case reports on affected children with broad clinical presentation and histologically proven, are available [10]. In the child studied, histopathologic examination including microscopic analysis with immunohistochemical reactions revealed lymphoplasmacytic infiltrates, storiform fibrosis, obliterative vascular lesions as well as IgG4:IgG ratio ≥40 % and IgG4-positive plasma cell numbers per HPF (high-power field) ≥10, meeting the criteria according to the Consensus Statement on Pathology of IgG4-RD [11]. Despite well-defined histological features of IgG4-RD in different unrelated organs, important questions need to be addressed about the exact role of IgG4, which is in general an antiinflammatory isotype [12, 13], in the immunopathology of this condition, the potential self or non-self antigens driving the immune response and the importance of atopy and hyperimmunoglobulinemia E [14, 15]. In our patient we performed a broad multidirectional differential diagnostic tests evaluating the immune response, allergy, autoimmunization and infections, showing positive Epstein-Barr virus (EBV)-DNA and abnormalities in adaptive immune response such as increased values of CD8+ T lymph cells and decreased values of CD4+ T lymph cells accompanied by features of defective B lymph cell maturation, with decreased formation in the switched and non-switched memory cells. EBV is an ubiquitous virus in humans and can remain latent throughout life, but in immunocompromised children it can reactivate and a correlation between EBV-DNA load in peripheral blood and severity of clinical manifestations was observed [16]. However, whether there is a causal relationship between an EBV infection as the source of a potential target antigen for IgG4 antibodies and defective collaboration between B and T lymph cells, histopathological features with plasmablast expansion remains to be elucidated [17]. Furthermore, clinicopathological correlations between morphological solid nodular, bronchovascular or alveolar interstitial types of pulmonary lesions and potential infectious, autoimmune, allergic and toxic factors remain to be determined [18–20]. Further studies are required in children with pulmonary presentation of IgG4-RD to better understand pathogenesis of this condition, possible immunological or infectious triggering factors, and finally, to determine pediatric patient-targeted therapeutic interventions.

## Abbreviations

AFP, alpha-fetoprotein; C, complement; CD, cluster of differentiation; CEA, carcinoembryonic antigen; CRP, C-reactive protein; CT, computed tomography; CXR, chest X-ray; DNA, deoxyribonucleic acid; EBV, Epstein-Barr virus; EMA, epithelial membrane antigen; EvG, Elastica van Gieson; HPF, high-power field; Ig, immunoglobulin; IgG4-RD, immunoglobulin G4-related disease; IHC, immunohistochemistry; SMA, smooth muscle actin; VATS, video-assissted thoracoscopy; VMA, vanillylmandelic acid; VRT, volume-rendering technique; WT, Wilm’s tumor; β-HCG, beta-human chorionic gonadotropin

## Additional file

Additional file 1: Timeline. (PPTX 37 kb)
