# Supplementary material for: Pulmonary manifestation of immunoglobulin G4-related disease in a 7-year-old immunodeficient boy with Epstein-Barr virus infection: a case report
Source: Ital J Pediatr. 2016 Jun 8;42:58. doi: 10.1186/s13052-016-0269-0 (PMC4898369; doi:10.1186/s13052-016-0269-0)
Supplement: Additional file 1: — Timeline. (PPTX 37 kb) [file 13052_2016_269_MOESM1_ESM.pptx]

## Slide 1
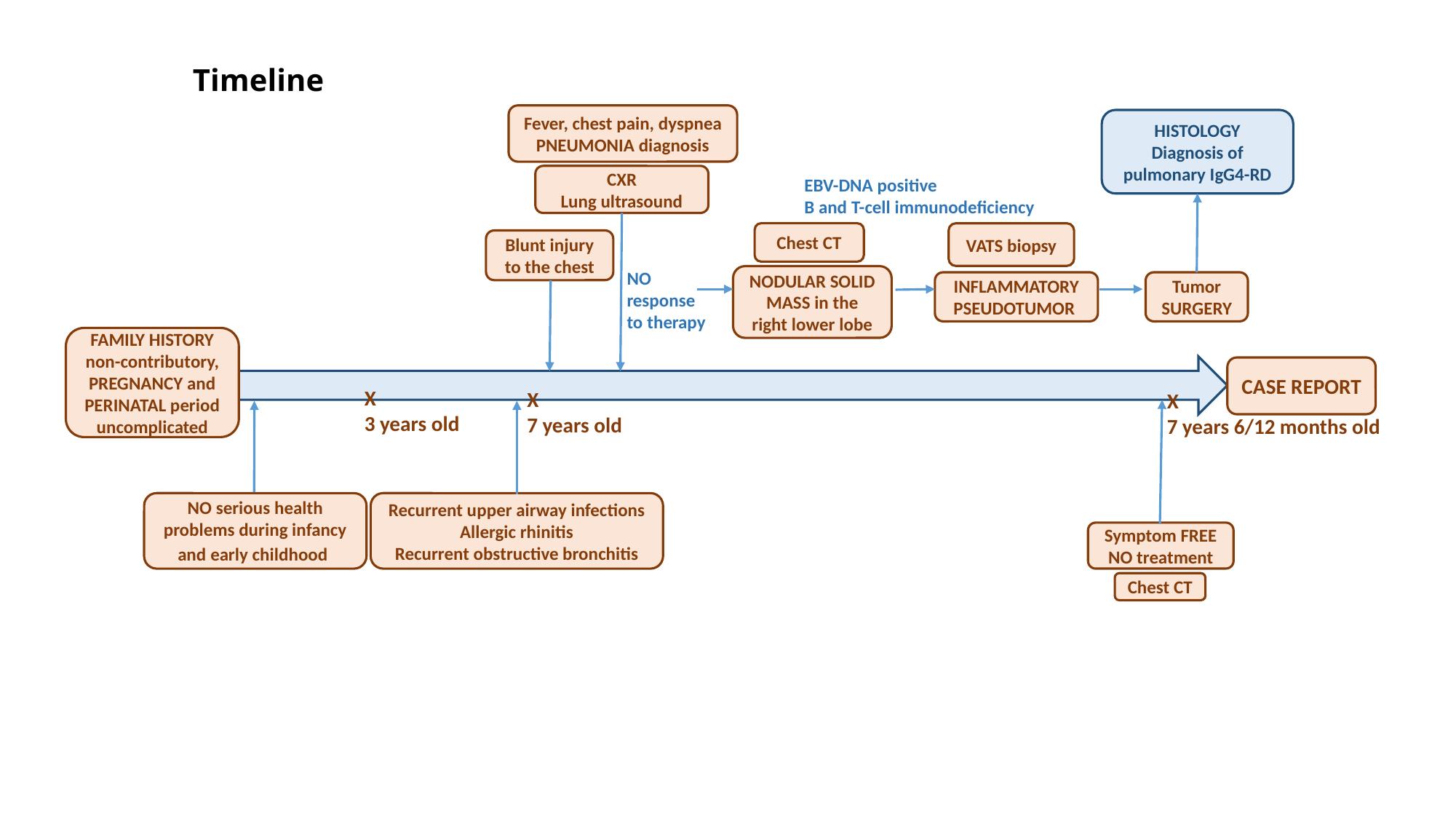

# Timeline
Fever, chest pain, dyspnea
PNEUMONIA diagnosis
HISTOLOGY
Diagnosis of pulmonary IgG4-RD
CXR
Lung ultrasound
EBV-DNA positive
B and T-cell immunodeficiency
Chest CT
VATS biopsy
Blunt injury to the chest
NO response to therapy
NODULAR SOLID MASS in the right lower lobe
INFLAMMATORY PSEUDOTUMOR
Tumor SURGERY
FAMILY HISTORY non-contributory,
PREGNANCY and PERINATAL period uncomplicated
CASE REPORT
X
3 years old
X
7 years old
X
7 years 6/12 months old
NO serious health problems during infancy and early childhood
Recurrent upper airway infections
Allergic rhinitis
Recurrent obstructive bronchitis
Symptom FREE
NO treatment
Chest CT
